# Stimulation of the farnesoid X receptor promotes M2 macrophage polarization

**DOI:** 10.3389/fimmu.2023.1065790

**Published:** 2023-01-27

**Authors:** Thiranut Jaroonwitchawan, Hideki Arimochi, Yuki Sasaki, Chieko Ishifune, Hiroyuki Kondo, Kunihiro Otsuka, Shin-ichi Tsukumo, Koji Yasutomo

**Affiliations:** ^1^ Department of Immunology and Parasitology, Graduate School of Medicine, Tokushima University, Tokushima, Japan; ^2^ Department of Interdisciplinary Research on Medicine and Photonics, Institute of Post-LED Photonics, Tokushima University, Tokushima, Japan; ^3^ The Research Cluster Program on Immunological Diseases, Tokushima University, Tokushima, Japan

**Keywords:** farnesoid X receptor, macrophage, cell differentiation, inflammation, tissue repair

## Abstract

FXR is a key molecule that modulates anti-inflammatory activity in the intestinal-liver axis. Although FXR has pleiotropic functions including regulation of liver inflammation and activation of macrophages, it remains unclear whether it is involved in macrophage polarization. In this paper we demonstrated that stimulation of macrophages derived from the bone marrow using an FXR agonist activated polarization toward M2 but not M1 macrophages. The treatment of mice with chitin skewed macrophage polarization towards M2 macrophages, while co-treatment with an FXR agonist further promoted the polarization toward M2 macrophages *in vivo*. This skewed polarization towards M2 macrophages by an FXR agonist was accompanied by increased expression of signaling molecules related to the retinoic acid receptor. Inhibition of the retinoic acid receptor suppressed FXR agonist-mediated M2 macrophage polarization, indicating that this polarization was, at least, partly dependent on the retinoic acid receptor pathway. These data demonstrate that FXR has a role in polarization toward M2 macrophages and suggest a possible therapeutic potential of FXR agonists in M2 macrophage-related conditions.

## Introduction

The macrophage is a key player in inflammatory processes, as it initially reacts with antigens, then digests them, resulting in the release of cytokines and chemokines that activate and recruit other immune cells (1–4). During the inflammatory process, macrophages are polarized into proinflammatory M1 macrophages that initiate adaptive immunity and tissue inflammation (4–7). Overactivation of M1 macrophages causes tissue damage and impairs the wound healing process. In contrast, M2 macrophages prevent tissue fibrosis after damage and initiate tissue regeneration and remodeling (4). M2 macrophages are also important in the response to helminth and fungal infections. Chitin is a component of fungi and an exoskeleton component of arthropods. Previous research suggests that administration of chitin in mice increases macrophage infiltration into the peritoneal cavity and induces activation of M2 macrophages (8, 9). Therefore, chitin-mediated M2 macrophage activation is useful for studying *in vivo* activation of these cells. The balance between tissue inflammation and repair offers a solution to prevent an unwanted amplification loop within the inflammation pathway. Both intrinsic and extrinsic cellular factors have been reported to be generally involved in determining macrophage polarization under inflammation conditions (5, 6).

The farnesoid X receptor (FXR, gene name: *Nr1h4*) is highly expressed in the liver and intestine, and its primary function is to act as a receptor for bile acid and also to orchestrate homeostasis (10–12). Activation of FXR induces a series of downstream pathways, which in turn, protect against inflammation, mainly in the gastrointestinal tract-liver axis (12, 13). A loss of function associated with a mutation in human *NR1H4* leads to progressive liver disease and high cholesterol levels, suggesting that FXR may contribute to liver disease (14). Several recent studies in experimental models have provided evidence that activation of the FXR signal has a protective role in inflammation by modulating bile acid homeostasis, intestinal integrity, and liver injury. For instance, FXR-deficient mice were shown to develop more severe liver inflammation, after administration of LPS or Con A-induced hepatitis (15, 16). Activation of FXR by an agonist was also shown to have a mitigating effect in the DSS-induced colitis model (17). Although several papers reported that FXR is involved in macrophage activation (18, 19), it remains to be determined whether FXR contributes to polarization of M1 and M2 macrophages. Therefore, we investigated the roles of FXR in macrophages, in which inflammatory properties are executed primarily *via* M1/M2 macrophage polarization.

In this paper we investigated whether FXR had a role in M1/M2 macrophage polarization and showed that an FXR agonist promoted M2 but not M1 polarization. Furthermore, the skewed polarization towards M2 macrophages was accompanied by increased expression of molecules related to retinoic acid receptor signaling. Inhibition of the retinoic acid pathway suppressed FXR agonist-mediated M2 macrophage polarization, indicating that FXR stimulates M2 macrophage polarization *via* the retinoic acid pathway. These data demonstrate that FXR contributes to M2 macrophage polarization and indicates that an FXR agonist has potential benefit to promote M2 macrophage-mediated tissue repair and remodeling.

## Material and methods

### Mice

C57BL6/J mice were purchased from Japan SLC (Shizuoka, Japan). All animal experiments were approved by the animal research committee of Tokushima University.

### Purification of immune cells

A spleen was excised and then smeared and filtered through a 70 μm filter. A single cell suspension was prepared and washed with washing buffer. Cell isolation procedures were performed according to the manufacturer’s instructions. CD19^+^ B cells were isolated first using CD19^+^ microbeads (Miltenyi Biotec, Bergisch Gladbach, Germany), with the negative fraction then used to enrich for CD3^+^ T cells using Pan T Cell Isolation (Miltenyi Biotec). The CD11c^+^ cells were labelled magnetically with CD11c^+^ ultrapure MicroBeads (Miltenyi Biotec). After sorting, the remaining negative fraction was separated to obtain CD11b^+^ cells using CD11b MicroBeads (Miltenyi Biotec) and then subjected to column separation. The purity of each immune cell type was assessed by flow cytometry.

### Preparation of macrophages

Male C57BL6/J mice (10-weeks-old) were used to prepare peritoneal macrophages and bone marrow derived macrophages (BMDMs). The peritoneal macrophages were obtained by flushing the peritoneal cavity two times with 5 mL of ice-cold culture medium containing RPMI-1640 (Nacalai Tesque, Kyoto, Japan), 10% FBS, 0.1% 2-mercaptoethanol (Thermo Fisher Scientific, Waltham, MA, USA), and 1% penicillin-streptomycin mixed solution (Nacalai Tesque) using a 25-gauge needle. The peritoneal fluid collected was poured into a 100 mm dish, followed by incubation for 4 hr in a CO_2_ incubator to isolate the cells attached to the bottom of the dish. After incubation, the dish was washed two times with 10 mL of pre-warmed culture medium, with the attached cells then collected by gentle scraping into 10 mL of culture medium for use in the experiments as peritoneal macrophages. For preparation of BMDMs, bone marrow cells were collected from femurs and tibias by flushing these bones with 10 mL of culture medium. After lysis of the red blood cells with 2 mL of hypotonic ammonium chloride solution, the cells were collected by centrifugation at 500 x g for 5 min at 4°C, and then cultivated for 7 d in 10 mL of culture medium containing recombinant mouse M-CSF (Pepro Tech, Cranbury, NJ, USA) at a concentration of 10 ng/mL.

### BMDM polarization and treatment with GW4064 and LE540

In order to initiate polarization towards M1 macrophages, the BMDMs were incubated for 24 hr in culture medium containing recombinant mouse IFNγ (Pepro Tech) and LPS (Sigma-Aldrich, St. Louis, MO, USA) at concentrations of 25 ng/mL and 2.5 µg/mL, respectively. M2 macrophages were generated by cultivation of BMDMs with 10 ng/mL of recombinant mouse IL-4 (Pepro Tech) for 24 hr. In some experiments, GW4064 (Sigma-Aldrich, EC50 = 15 nM) and LE540 (FUJIFILM Wako Pure Chemical, Osaka, Japan) dissolved in DMSO were diluted with the culture medium and then added into the culture medium during M1 and M2 induction.

### RNA extraction and quantitative RT-PCR

RNA was extracted from 1 x 10^6^ of macrophages with RNAiso Plus (Takara Bio, Shiga, Japan), according to the protocol provided by the manufacturer. The quality and concentration of the extracted RNA were determined using a Nanodrop 2000 spectrophotometer (Thermo Fisher Scientific). Complementary DNA was generated from 120 ng of the extracted RNA using the ReverTra Ace^®^ qPCR RT Master Mix with gDNA Remover (Toyobo, Osaka, Japan) in accordance with the manufacturer’s protocol. Quantitative RT-PCR was conducted using the QuantStudio^®^ 5 real-time PCR system (Thermo Fisher Scientific) and Applied Biosystems SYBR Green Master Mixes (Thermo Fisher Scientific). Relative gene expression level was estimated by normalizing the expression of each target gene to that of the hypoxanthine phosphoribosyl transferase (HPRT) gene. The sequences of the primer sets used for qPCR in this study are listed in **Supplementary Table**.

### Western blotting

BMDMs (2×10^6^ cells) were seeded in six-well plates and cultured for 48 hr under M2 conditions with or without GW4064 and LE540. The cells were lysed with RIPA buffer (Nacalai Tesque) containing protease inhibitors (Roche, Basel, Switzerland). The lysates were resolved by SDS-PAGE and the blots incubated with anti-ARG-1 (E2, Santa Cruz biotech, Dallas, TX, USA) or anti-actin (A2066, Sigma-Aldrich) antibodies, followed by incubation with HRP-conjugated goat anti-mouse IgG antibody (Merk, Darmstadt, Germany). The bands were detected using ECL Prime Chemiluminescent Substrate (GE Healthcare, Chicago, IL, USA) and the ImageQuant LAS-4000 Mini System (GE Healthcare).

### Chitin treatment

Female C57BL/6 mice were injected intraperitoneally with 50 µg of chitin particles from shrimp shells (C9752, Sigma-Aldrich) for 3 hr to induce M2 macrophages, followed by the first dosage of GW4064 (30 mg/kg) and 2 mg/kg LE540 or vehicle control. A second dose of GW4064 and LE540 at the same concentration as the first was then administered and the peritoneal macrophages collected for analysis.

### Flow cytometric analysis

BMDMs (1 x 10^6^ cells per sample) were treated with 5 or 20 μM GW4064 for 3 d in 6-well plates under the conditions used to induce M2 macrophages. The same amount of DMSO with GW4064 was added to the M2-inducing or neutral culture systems to prepare 0 μM GW4064 samples. After treatment of the cells, each well was washed 2 times with 1 mL of ice-cold PBS and the cells then collected by gentle scraping in PBS, followed by centrifugation at 500 x g for 5 min at 4°C. The collected cells were treated with 2.4G2 antibody at 4°C for 15 min, washed with FACS buffer made of PBS, 2% FBS, and 0.05% sodium azide, and then stained at 4°C for 15 min with either propidium iodide (PI), PE-anti CD11c (BioLegend, clone N418), APC-anti CD206 (BioLegend, clone C068C2), APC-Cy7-anti CD11b (BioLegend, clone M1/70), or Pacific blue-anti F4/80 (BioLegend, clone BM8) in FACS buffer. Flow cytometric analysis was conducted using the BD FACSCanto II system (Becton, Dickinson and Company, Franklin Lakes, NJ, USA) by acquiring at least 10,000 events for each sample. The data obtained was analyzed using FACSDiva software (BD Biosciences, Franklin Lakes, NJ, USA) to calculate the mean fluorescence intensity (MFI) of CD206 expressed on the surface of PI^-^ CD11b^+^ F4/80^+^ live macrophages.

### Microarray analysis

Total RNA was extracted from 10^6^ of BMDMs treated for 24 hr with 10 μM GW4064 or DMSO under M2 conditions using the ReliaPrep RNA Cell Miniprep system (Promega, Madison, WI, USA) in accordance with the manufacturer’s protocol. RNA integrity was detected using an Agilent 2100 Bioanalyzer (Agilent Technologies, Santa Clara, CA, USA), while microarray analysis was performed using a 44K Whole Genome mRNA microarray system (Agilent Technologies). Biological process annotation and pathway analysis with normalized and filtered data was performed with GeneSpring 14 software.

The microarray results are available in the NCBI GEO website (accession number: GSE216995).

### Statistical analysis

The representative data of the results were expressed as mean +/- SD. One way ANOVA was performed for multiple comparisons, while Student’s t test was used for comparisons between two groups.

## Results

### Activation of FXR in macrophages contributed to promotion of M2 macrophages

**Figure 1:**
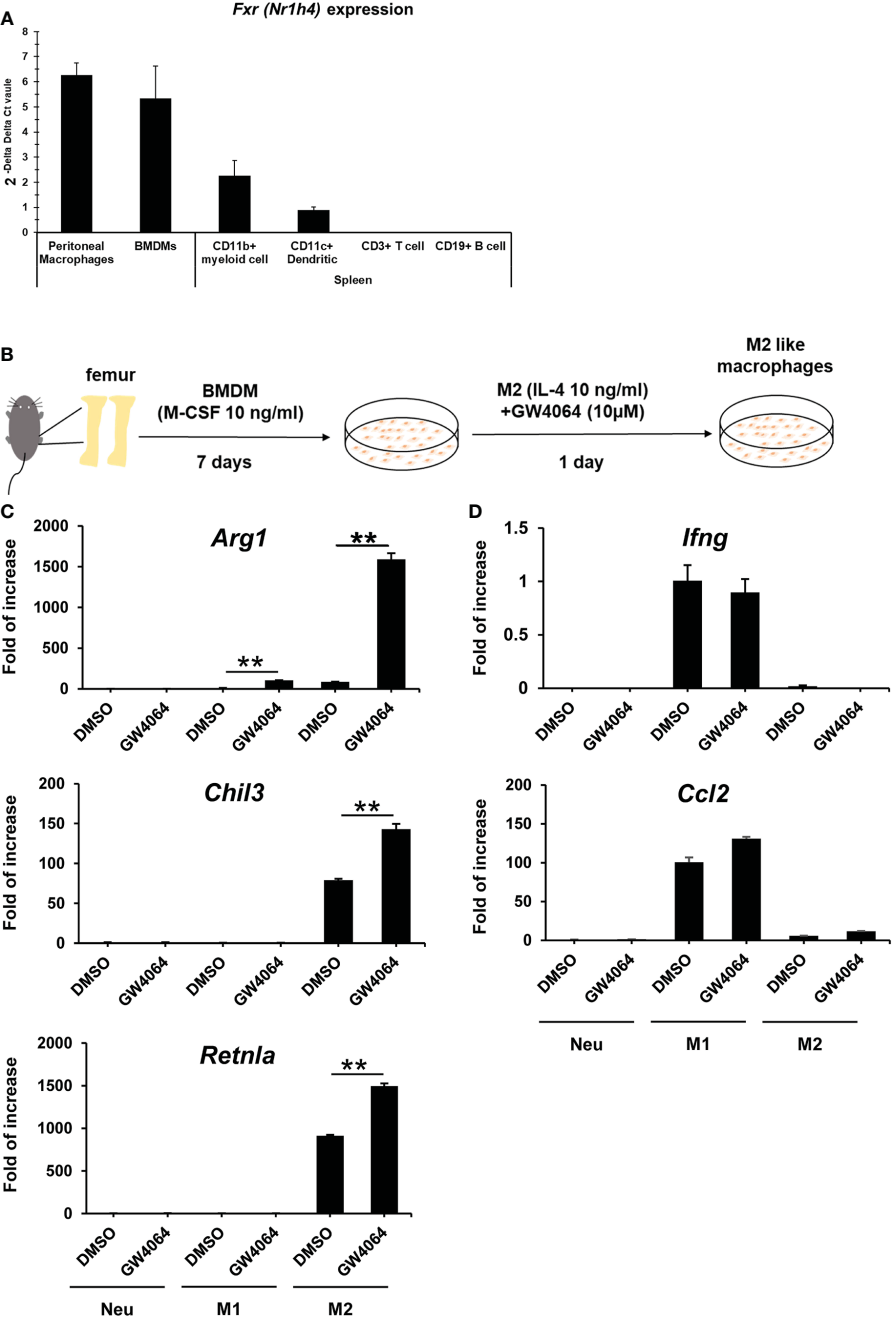
Activation of FXR in macrophages by GW4064 treatment selectively enhances M2- but not M1-associated genes. **(A)** mRNAs from peritoneal macrophages, BMDMs, splenic myeloid cells, dendritic cells, T cells, and B cells were extracted and converted to cDNA. Relative FXR mRNA gene (*Nr1h4*) expression in these cells was determined by real-time PCR. **(B)** Schematic representation of the M2 macrophage polarization protocol. Bone marrow cells were isolated from mice femurs and cultivated for 7 d in the presence of recombinant M-CSF to become BMDMs. M2 polarization was then induced for 1 d using 10 ng/mL of recombinant IL-4. **(C)** BMDMs were treated with 10 µM of GW4064 under M1 or M2 macrophage-inducing conditions and left untreated as a Neu condition for 1 d. RT-qPCR analysis for M2-related genes was then conducted. **(D)** Real-time PCR analysis for M1-related genes was performed by using the same samples used in **(C)**. Each experiment was performed in triplicate and repeated three times. The results are representative data, expressed as mean +/- standard deviation. Analysis of variance (ANOVA) was used for the statistical analysis. **P < 0.01. *Nr1h4*, nuclear receptor subfamily 1 group H member 4; M-CSF, macrophage colony-stimulating factor; Neu, neutral.

FXR is expressed in multiple tissues, particularly the liver and intestine (20). To investigate the roles of FXR in macrophages, we first examined *Nr1h4* mRNA gene expression in immune cells. We sorted CD3^+^ T cells, CD19^+^ B cells, CD11c^+^ dendritic cells and CD11b^+^ myeloid cells from B6 spleens, collected peritoneal CD11b^+^F4/80^+^ macrophages, and prepared BMDMs. In splenic CD11b^+^ myeloid cells, mRNA *Nr1h4* expression was moderately lower than that observed in peritoneal macrophages and BMDMs, whereas *Nr1h4* expression was detected only rarely in the T and B cell lineages (**Figure 1A**). This suggested that FXR in macrophages may be functional and relevant in BMDMs and peritoneal macrophages. We first set up an experimental protocol for inducing M2 macrophages using BMDMs, which was started using a standard protocol for BMDM differentiation supplemented with recombinant mouse M-CSF (**Figure 1B**). The M2 macrophages were induced by 10 ng/mL of IL-4 for 1 d with or without 10 µM of GW4064. (**Figure 1B**). The treatment with GW4064 did not affect cell survival of bone marrow cells. Treatment with GW4064 increased mRNA expression of the key M2 macrophage genes, *Arg1, Chil3*, and *Retnla*, whereas GW4064 did not change the expression of M2 macrophage-related genes under M1 conditions, except for *Arg1* that was increased slightly compared to that observed in neutral conditions (**Figure 1C**). In contrast, GW4064 treatment did not induce the M1-related genes, *Ccl2 and Ifng*, under M2 macrophage polarization conditions (**Figure 1D**).

### Activation of FXR in BMDM promoted M2 macrophages

**Figure 2:**
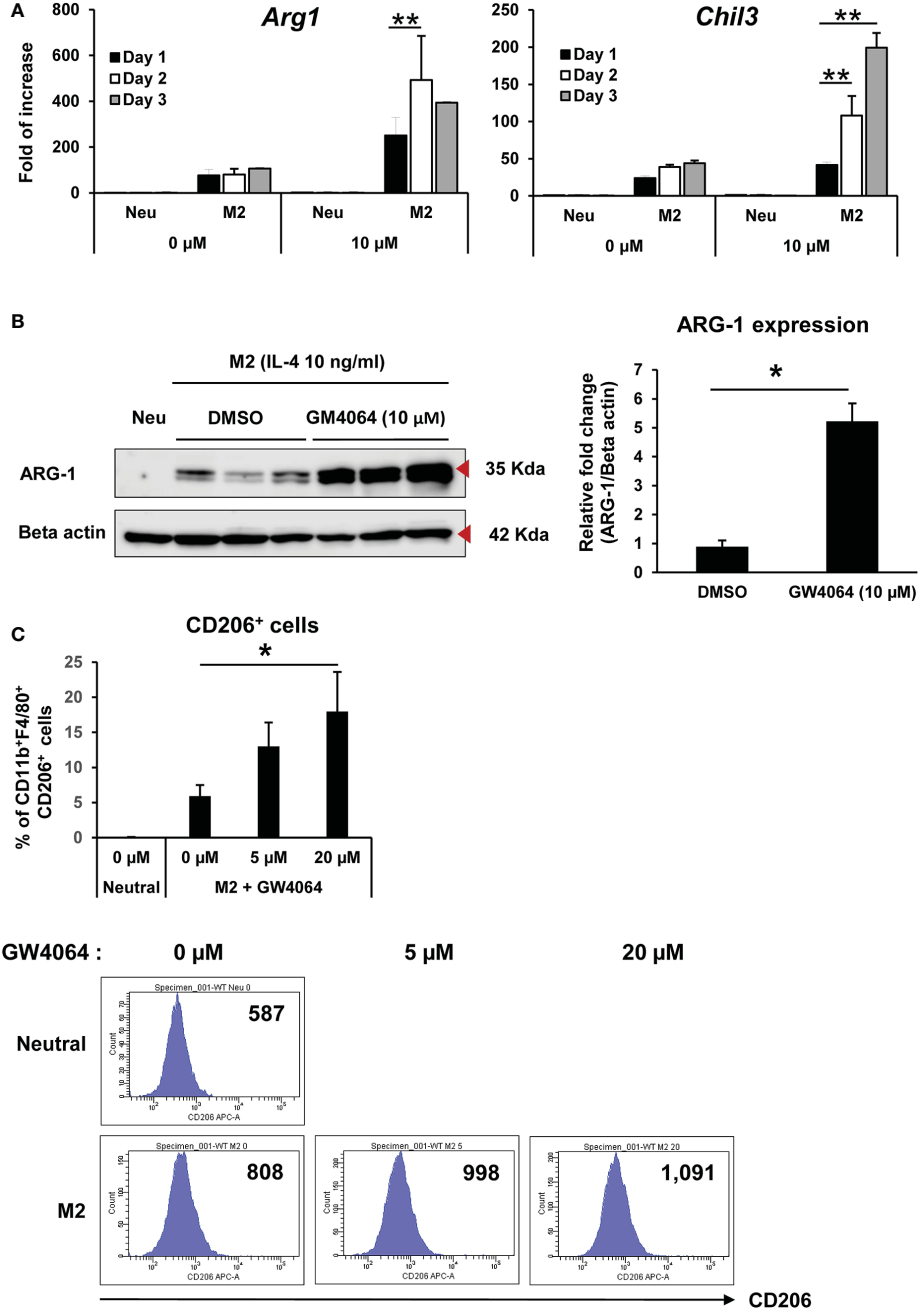
Activation of FXR with GW4064 increased expression of M2-associated markers. **(A)** BMDMs were treated with or without 10 µM of GW4064 under the M2 condition from 1 to 3 d, and left untreated as a Neu condition and M2-associated gene expression then measured by real-time PCR. **(B)** ARG-1 protein (35 kDa) expression was examined by immunoblotting on day 2 of GW4064 treatment under M2 conditions, with relative ARG-1 expression normalized to β-actin (42 kDa) expression (left panel). The graph shows the relative expression of ARG-1 protein in GW4064-treated cells compared to that of the vehicle control group (right panel). **(C)** BMDMs were treated with 0, 5, or 20 µM of GW4064 under the M2 condition for 3 d. Flow cytometry analysis of CD11b^+^ F4/80^+^ CD206^+^ and CD206 expression on the cell surface was performed, with the results expressed as the percentage of CD206 in CD11b^+^ F4/80^+^ cells (upper panel) and MFI (lower panel). Each experiment was carried out in triplicate and repeated three times. The results of the representative data are shown as mean +/- SD. Analysis of variance (ANOVA) and the Student’s t test were used for the statistical analyses. MFI: mean fluorescence intensity. *P < 0.05, **P < 0.01.

We next analyzed the time and dose course of M2 macrophage polarization induced by an FXR agonist. Treatment with GW4064 increased *Arg1* and *Chil3* mRNA expression in a time-dependent manner (**Figure 2A**). In addition, GW4064 treatment clearly increased ARG-1 protein under M2 macrophage polarization conditions compared with that observed in the control (**Figure 2B**). In accordance with the increase in ARG-1, GW4064-treated BMDM under M2 conditions showed a significant increase in the percentage of CD11b^+^ F4/80^+^ CD206^+^ (M2) cells at a dose of 20 µM of GW4064, with the mean fluorescence intensity of CD206, a marker for M2 macrophages, upregulated by increasing doses of GW4064 (**Figure 2C**). The gating strategy in **Figure 2C** is shown in **supplementary Figure 1**. Collectively, these data indicated that GW4064-mediated FXR activation selectively promoted M2 macrophages.

### Retinol metabolism was upregulated following FXR activation in M2 macrophages

**Figure 3:**
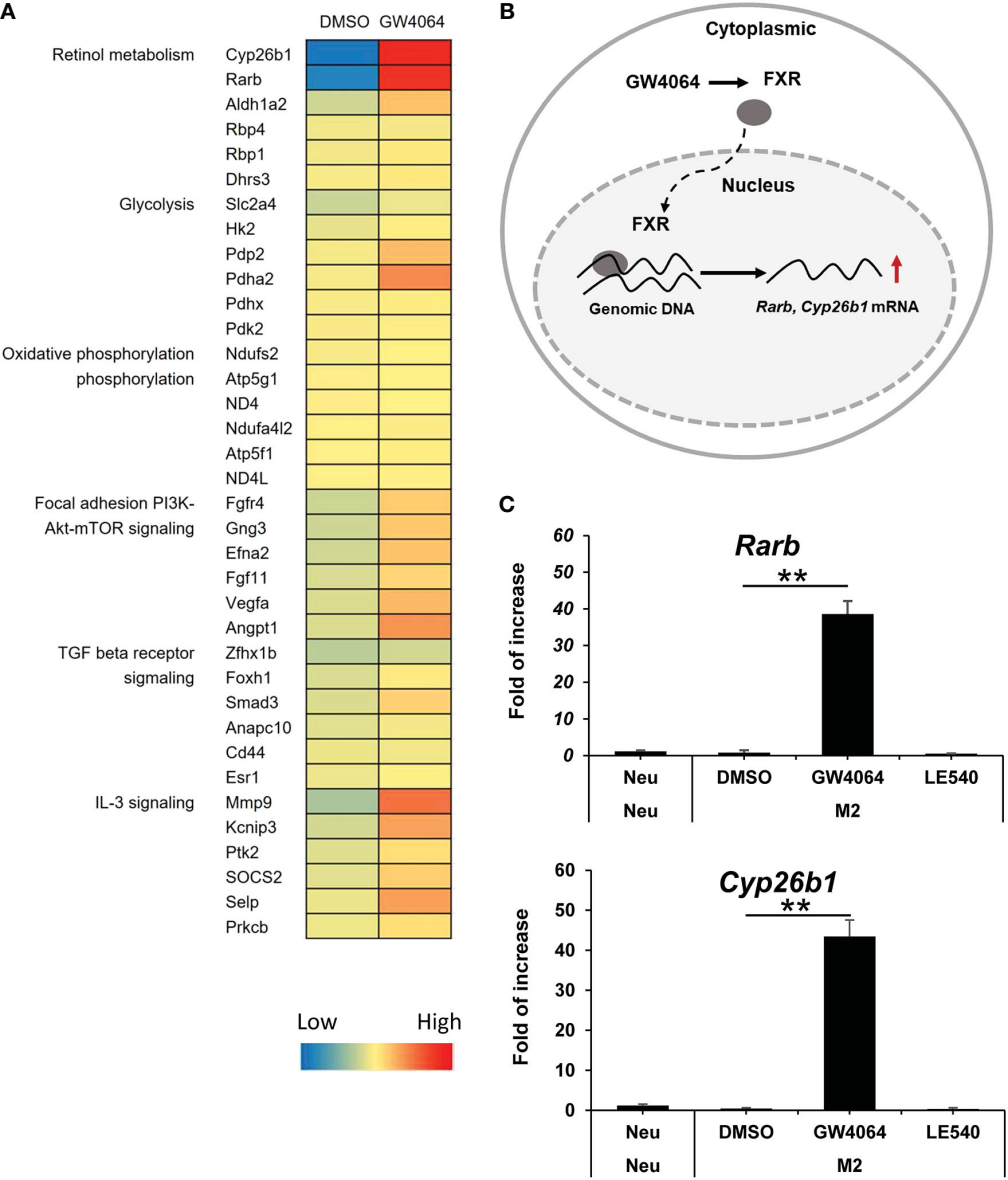
Rarb and Cyb26b1 expression was increased by FXR activation with GW4064 in M2 macrophages. **(A)** BMDMs were treated with DMSO or 10 µM of GW4064 under M2-inducing conditions for 1 d. The mRNAs were isolated and checked for integrity and a DNA micro array then used to profile differential gene expression. The heatmap analysis shows profiling of gene alterations according to biological process annotations and pathway analysis. **(B)** A diagram showing that RARB and CYP26B1 are included in the retinoic acid signaling pathway. **(C)** Real-time PCR was performed to assess *Rarb* and *Cyb26b1* mRNA gene expression in BMDMs treated with DMSO, 10 µM of GW4064, or 2 µM of LE540 under M2-inducing conditions for 1 d and left untreated as a Neu condition. Each experiment was performed in triplicate and repeated three times. The results of the representative data are shown as mean +/- SD. Analysis of variance (ANOVA) was used for the statistical analysis. ** *P* < 0.01. *Rarb*: Retinoic acid receptor beta; *Cyp26b1*: Cytochrome P450 family 26 subfamily B member 1.

Because FXR activation induced M2 polarization but not M1 polarization, we next investigated how FXR activation contributed to M2 macrophages. To address this point, we first treated BMDMs under M2 conditions in the presence of GW4064 or its vehicle control for 1 d and then performed DNA microarrays to profile the altered genes. As shown in **Figure 3A**, alternative gene expression shown in heatmaps and pathway analysis provided a comprehensive view of the genes involved in metabolic pathways including glycolysis and focal adhesion of PI3K-Akt mTOR under GW4064 treatment. These signaling pathways were essential for normal cell survival and differentiation. The list of top-ranking genes showed a significant increase in retinol metabolism. As shown in **Figure 3B**, DNA microarray analysis revealed that *Rarb* and *Cyp26b1* were upregulated when FXR was activated under M2 conditions. As a consequence, we used qPCR to evaluate alterations in mRNA expression of genes involved in retinal metabolism including *Rarb* and *Cyp26b1.* Under M2 macrophage polarization conditions, GW4064 treatment increased both *Rarb* and *Cyp26b1* mRNA expression, but decreased it in the presence of LE540, an inhibitor of the retinoic acid receptor (**Figure 3C**). These findings suggested that the retinoic acid receptor might be, at least partly, involved in FXR-activated M2 macrophage polarization.

### Inhibition of the retinoic acid receptor influenced FXR activation in M2 macrophages

**Figure 4:**
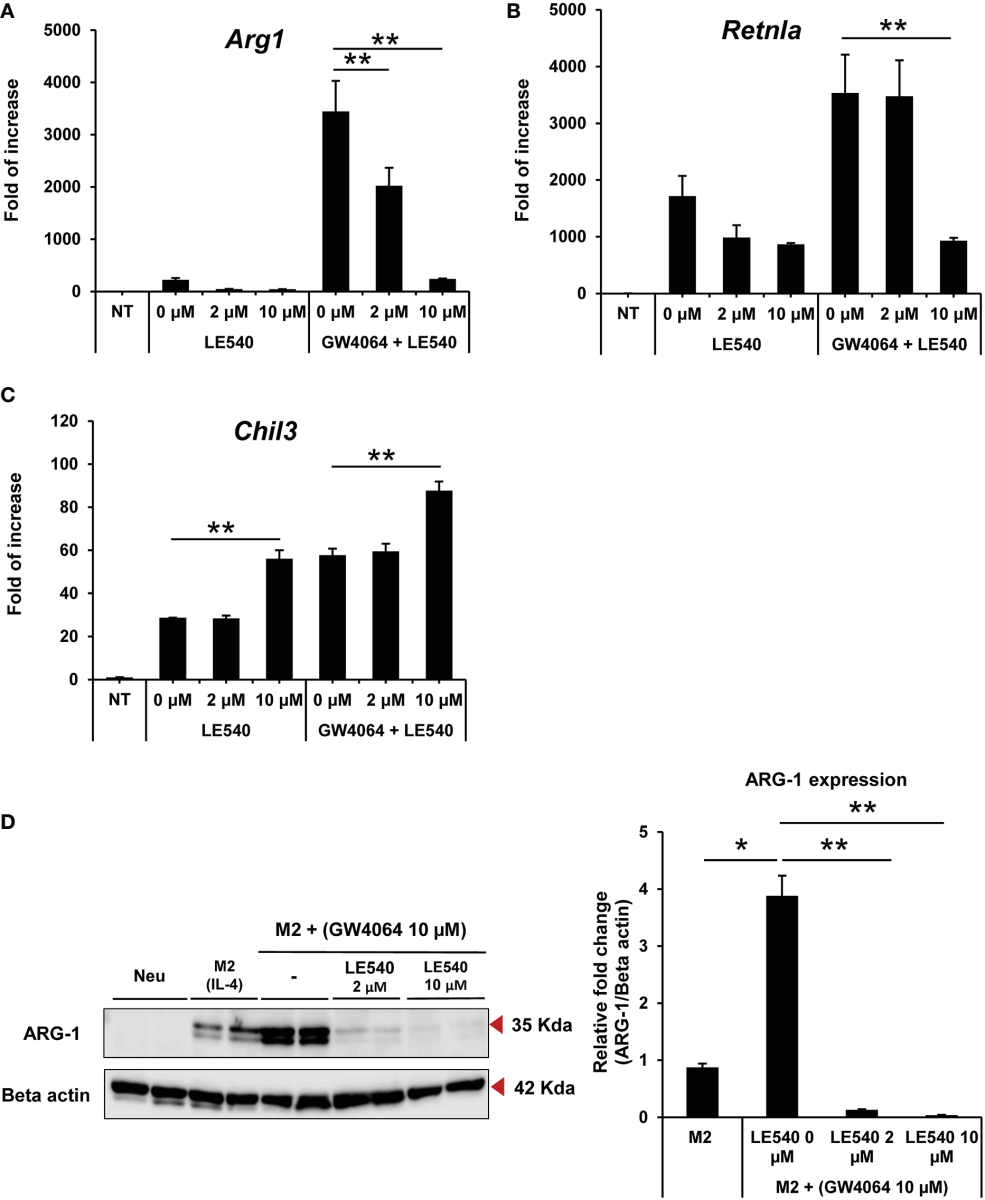
Inhibition of the retinoic acid receptor by LE540 reduced expression of M2-related markers induced by GW4064-mediated FXR activation. BMDMs were treated with 2 and 10 μM of LE540 with or without 10 μM of GW4064 under M2 conditions for 1 d. Messenger RNA gene expression of Arg1 **(A)**, Retnla **(B)** and Chil3 **(C)** was evaluated by RT-qPCR. **(D)** Immunoblotting analysis of ARG-1 (35 kDa) expression in BMDMs treated with 2 and 10 μM of LE540 with or without 10 μM of GW4064 under M2 conditions for 2 d and left untreated as a Neu condition (left panel). The expression level of ARG-1 was normalized to β-actin (42 kDa) expression, with the graph showing the relative expression of ARG-1 in BMDMs treated with or without LE540 in the presence of GW4064 compared to that observed with the control M2 condition (right panel). Each experiment was performed in triplicate and repeated three times. The results of the representative data are shown as mean +/- SD. Analysis of variance (ANOVA) was used for the statistical analysis. *P < 0.05, **P < 0.01.

We next investigated whether altered retinoic acid signaling by the retinoic receptor inhibitor (LE540) contributed to GW4064-induced polarization towards M2 macrophages. The expression of *Arg1* (**Figure 4A**) and *Retnla* (**Figure 4B**) was decreased markedly in M2 macrophages at 2 and 10 µM of LE540, respectively, whereas expression of *Chil3* remained unaltered (**Figure 4C**). The treatment of cells with LE540 did not affect cell survival of macrophages. The inhibition of ARG-1 expression was validated at the protein level, with this expression markedly reduced in the presence of LE540 and GM4064 in a dose-dependent manner (**Figure 4D**). These findings indicated that the retinoic acid receptor complements FXR activation in M2 macrophages by increasing ARG-1.

### Retinoic acid receptor inhibitor reduced FXR-mediated M2 macrophages in chitin-treated mice

**Figure 5:**
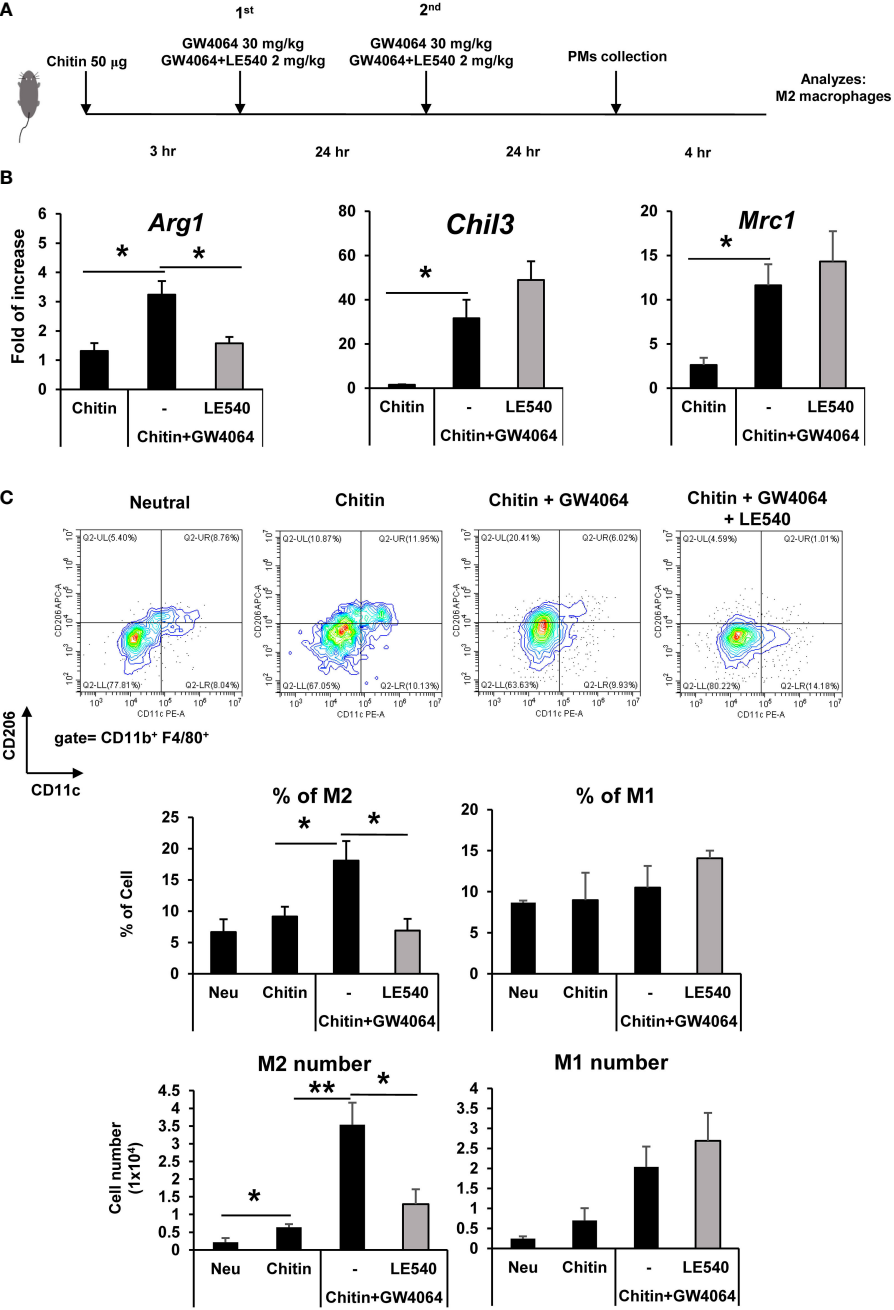
Administration of a retinoic acid receptor inhibitor reduced GW4064-enhanced M2 macrophages in chitin-treated mice. **(A)** The schematic protocol to induce M2 peritoneal macrophages by injection of chitin particles and assess the effects of GW4064 and LE540 on M2 macrophage induction *in vivo*. The mice received an intraperitoneal injection of 50 µg of chitin to prime M2 induction for 3 hr and then either 30 mg/kg bodyweight of GW4064 or GW4064 together with 2 mg/kg bodyweight of LE540 were administered two times within a 24 hr interval. **(B)** Peritoneal macrophages were collected for further analysis. Messenger RNA expression of M2-associated genes in collected peritoneal macrophages (PMs) was analyzed by real-time PCR. **(C)** Flow cytometric analysis of CD206^+^ (M2) and CD11c^+^ (M1) from CD11b^+^ F4/80^+^ PMs. The frequency and total numbers of each population are shown in the lower panel. Five mice were used in each experimental group, except for the control group Neu in which four mice were used. Each experiment was repeated two times independently (n=4-5 mice/group), and the result of the representative data shown as mean +/- SE. Analysis of variance (ANOVA) was used for the statistical analysis. *P < 0.05, **P < 0.01.

We analyzed whether the retinoic acid pathway played an important role in the polarization of FXR-mediated M2 macrophages *in vivo*. Chitin administration to mice was used to initiate M2 macrophage polarization and examine the function of GW4064 FXR activation in promoting this polarization. We established a protocol to induce macrophages to the M2 state *in vivo* by administering chitin particles to prime murine peritoneal macrophages (**Figure 5A**). This schematic protocol involved a mouse model in which chitin particles were injected intraperitoneally. After a second dose of GW4064, GW4064 and LE540 was administered, peritoneal macrophages were collected for analysis (**Figure 5A**). Administration of GW4064 upregulated M2-associated gene expression (*Arg1*, *Chil3*, and *Mrc1*) in peritoneal macrophages of chitin-treated mice (**Figure 5B**). GW4064 treatment increased the frequency and number of M2 macrophages (CD206^+^ CD11c^-^) in the chitin-treated group compared to control group but did not affect M1 macrophage (CD206^-^ CD11c^+^) polarization (**Figure 5C**). In addition to GW4064 treatment, administration of LE540 reduced the frequency and number of chitin and GW4064-mediated M2 macrophages (CD206^+^ CD11c^-^) compared to that observed in the LE540-untreated group (**Figure 5C**). Treatment with LE540 did not affect M1 macrophage (CD206^-^ CD11c^+^) polarization. Those data indicated that retinoic acid signaling was involved in FXR-mediated M2 macrophages *in vivo.*

## Discussion

Macrophages are involved in various aspects of tissue inflammation, with polarization towards M1 and M2 macrophages being one determinant for the outcome of inflammation (1, 6, 21). This study demonstrated that an FXR agonist, GW4064, promoted polarization toward M2 macrophages both *in vitro* and *in vivo*. In addition, polarization toward M2 macrophages induced by GW4064 treatment was accompanied by increased expression of molecules related to the retinoic acid pathway. Inhibition of retinoic acid was shown to suppress FXR-mediated polarization toward M2 macrophages. The study therefore provided evidence that FXR has a role in M2 macrophage polarization and suggests that GW4064 treatment may provide a novel therapeutic strategy for stimulating M2 macrophage-mediated tissue repair or remodeling.

Bile acids (BAs) are generated by hepatocytes and have a role in dietary lipid absorption and cholesterol homeostasis (22, 23). BAs bind to FXR, a member of the nuclear receptor super-family that has transcriptional activity. FXR binds to FXR response elements as a monomer or heterodimer in conjunction with the retinoid X receptor (RXR) and has pleiotropic functions including immune cell activation and lipid metabolism (13, 24). The current study demonstrated that stimulation of macrophages with an FXR agonist increased the activity of signaling molecules related to retinoic acid receptors and inhibited the retinoic acid pathway, resulting in at least partial suppression of M2 macrophage polarization. Retinoic acid receptors (RARs) are nuclear hormone receptors that contain α, β, and γ isotypes, and act as a binding partner for RXR (25). The question remains as to how FXR regulates the polarization of M2 macrophages through retinoic acid receptors. One possible explanation is that FXR contributes to increased expression of RARB, which strengthens retinoic acid receptor signaling. That idea is supported by a recent study that demonstrated retinoic acid promoted polarization of M2 macrophages (26). However, we cannot completely abrogate M2 macrophage polarization by inhibiting the retinoic acid pathway. This indicates that FXR may also regulate M2 macrophage polarization programs independent of the retinoic acid pathway. FXR may regulate M1 macrophage polarization independent of the M2 program.

We identified an M2 enhancement mechanism induced by a FXR agonist in BMDMs. Because BMDMs are naïve or less activated by the cytokine environment during the differentiation or activation processes compared to that obtained *in vivo* with other macrophages including peritoneal macrophages, we used BMDMs to study the mechanisms of the M2-enhancing effect caused by the FXR agonist *in vitro* system. We also showed that the M2-enhancing effect of GW4064 *in vitro* and in the *in vivo* chitin treatment model could enhance M2 polarization. Furthermore, the expression level of the *Fxr* gene was comparable in BMDMs and peritoneal macrophages. These results indicated that FXR activation may have a shared underlying mechanism in regulating M2 in both BMDMs and peritoneal macrophages.

The results showing that the expression of IL-4-mediated M2 related genes in BMDMs was further enhanced when treated together with a FXR agonist suggested signaling crosstalk between the FXR and IL-4 pathways. Study of the molecular mechanisms and the roles of signaling cross talk in M2 polarization would be worthwhile in future studies. Our findings also suggested that FXR activation in combination with retinoic acid signaling favored M2 macrophage polarization rather than mediating M1 polarization. In contrast, Murray et al. (19) demonstrated that nitrogen mustard (NM) generated both M1 and M2 in FXR-deficient mice leading to lung injury. This contradicts our findings that FXR activation favored M2 macrophage polarization rather than M1 polarization. This discrepancy may be explained by the different stimulator used in each experiment. Instead of NM, we conducted an experiment with IL-4 and chitin to induce M2 *in vitro* and *in vivo*, respectively. Stimulation of NM and chitin may induce distinct underlying mechanisms that influence the outcome of M1/M2 polarization in microenvironments. In addition, in the microenvironment of lung tissue, immune and non-immune cells might have a critical role of interacting with other cells to modulate M1/M2 polarization of macrophages.

We evaluated the involvement of RAR in FXR activation of M2 macrophages *in vivo*. LE540 had the opposite effect on Arg1 gene expression compared to that induced by other M2-related genes. This discrepancy might be explained by the fact that *Arg1* expression is dependent on the retinoic acid pathway whereas other M2-related genes such as *Chil3* are not. Our results also demonstrated that polarization of GW4064-mediated M2 macrophages was reduced by co-administration of the LE540 RAR antagonist. This result can be further explained by the fact that the retinoic acid receptor is involved in FXR-mediated polarization of M2 macrophages in mice treated with chitin particles. We cannot rule out the possibility that FXR or retinoic acid receptor signaling in non-macrophages cells indirectly regulates M2 macrophages in the peritoneal cavity because hepatocytes and intestine also strongly express FXR. In addition, a limitation of our study was that our experiments were conducted by hyperactivating FXR using the GW4064 agonist, which may induce FXR-independent signaling that currently has not yet been defined. In future experiments, FXR-deficient mice, tissue-specific FXR-deficient mice, or retinoic acid receptor-deficient mice would be valuable models for studying the roles of FXR and the retinoic acid receptor in macrophage polarization.

In conclusion, we demonstrated that an FXR agonist mediated M2 macrophage polarization both *in vitro* and *in vivo* and that this effect was, at least partly, dependent on the retinoic acid pathway. We propose that FXR agonists may be therapeutically useful for treating M2 macrophage-mediated tissue repair or remodeling.

## Data availability statement

The raw data supporting the conclusions of this article will be made available by the authors, without undue reservation.

## Ethics statement

The animal study was reviewed and approved by Animal Research committee in Tokushima University.

## Author contributions

TJ, HA, and KY conceived and designed the studies. TJ and HA performed and analyzed all the experiments. CI, YS, KO, HK, and S-IT analyzed the data. TJ, HA, and KY wrote the paper, and all authors reviewed the final version of the paper. KY supervised all the experiments. All authors contributed to the article and approved the submitted version.
