# Supplementary material for: Stimulation of the farnesoid X receptor promotes M2 macrophage polarization
Source: Front Immunol. 2023 Jan 27;14:1065790. doi: 10.3389/fimmu.2023.1065790 (PMC9911659; doi:10.3389/fimmu.2023.1065790)
Supplement: Supplementary file 1 [file DataSheet_1.pdf]

Supplementary Figure 1.

Gating strategy for detecting macrophages.

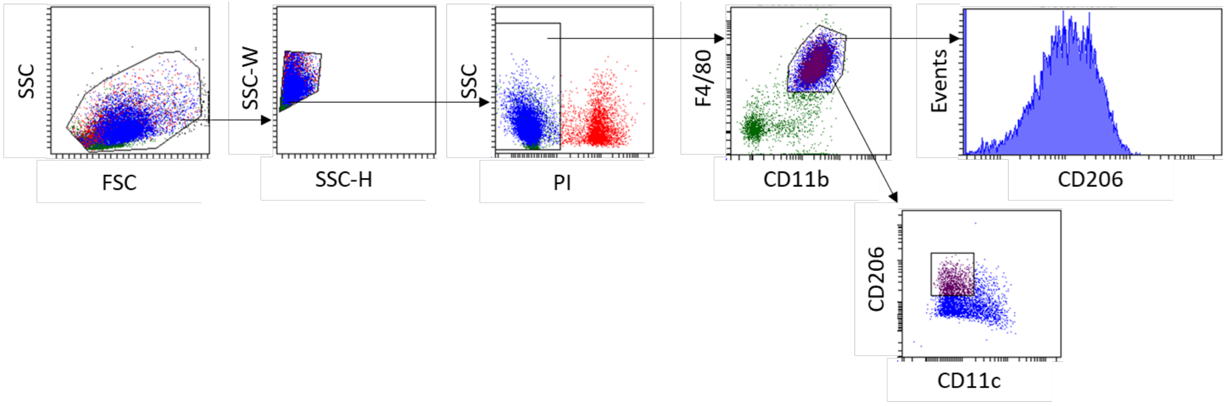

Cells were stained with several antibodies and analyzed by flow cytometer.

TABLE List of qPCR primer sequences

Gene	Forward primer (5'→ 3')	Reverse primer (5'→ 3')
Arg1	CACGGCAGTGGCTTTAACCT	CAGTCCCTGGCTTATGGTTACC
Ccl2	TTAAAAACCTGGATCGGAACCAA	GCATTAGCTTCAGATTTACGGGT
Chil3	GGGCTAAGGACAGGCCAATA	ATTCTGCATTCCAGCAAAGG
Cyp26b1	TCATCGGAGAGACTGGTCACT	GGTGCTCACTAGCTGGTGTTC
Hprt	CCAAAATGGTTAAGGTTGC	CCAGTTTCACTAATGACACAAC
Ifng	AGCGGCTGACTGAACTCAGATTGTAG	GTCACAGTTTTCAGCTGTATAGGG
Mrc1	TCCCTGCCTGTTTCTCCAACCA	TAAGCTTCGGCTCGTCAGCA
Nr1h4	CTTGATGTGCTACAAAAGCTGTG	ACTCTCCAAGACATCAGCATCTC
Rarb	CTGCTCAATCCATCGAGACAC	CTTGTCCTGGCAAACGAAGC
Retnla	ACTGCCTGTGCTTACTCGTTG	GCTGGGTTCTCCACCTCTTC
